# Determination of genetic predisposition to early breast cancer in women of Kazakh ethnicity

**DOI:** 10.18632/oncotarget.28518

**Published:** 2023-10-04

**Authors:** Gulnur Zhunussova, Nazgul Omarbayeva, Dilyara Kaidarova, Saltanat Abdikerim, Natalya Mit, Ilya Kisselev, Kanagat Yergali, Aigul Zhunussova, Tatyana Goncharova, Aliya Abdrakhmanova, Leyla Djansugurova

**Affiliations:** ^1^Laboratory of Molecular Genetics, Institute of Genetics and Physiology, Almaty 050060, Kazakhstan; ^2^Kazakh Institute of Oncology and Radiology, Almaty 050060, Kazakhstan; ^3^Al-Farabi Kazakh National University, Almaty 050060, Kazakhstan; ^4^Asfendiyarov Kazakh National Medical University, Almaty 050060, Kazakhstan

**Keywords:** early-onset breast cancer, triple negative breast cancer, next-generation sequencing, pathogenic variant, Kazakh population

## Abstract

Breast cancer (BC) is the most common type of cancer among women in Kazakhstan. To date, little data are available on the spectrum of genetic variation in Kazakh women with BC. We aimed to identify population-specific genetic markers associated with the risk of developing early-onset BC and test their association with clinical and prognostic factors. The study included 224 Kazakh women diagnosed with BC (≤40 age). Entire coding regions (>1700 exons) and the flanking noncoding regions of 94 cancer-associated genes were sequenced from blood DNA using MiSeq platform. We identified 38 unique pathogenic variants (PVs) in 13 different cancer-predisposing genes among 57 patients (25.4%), of which 6 variants were novel. In total, 12 of the 38 distinct PVs were detected recurrently, including *BRCA1* c.5266dup, c.5278-2del, and c.2T>C, and *BRCA2* c.9409dup and c.9253del that may be founder in this population. *BRCA1* carriers were significantly more likely to develop triple-negative BC (OR = 6.61, 95% CI 2.44–17.91, *p* = 0.0002) and have family history of BC (OR = 3.17, 95% CI 1.14–8.76, *p* = 0.03) compared to non-carriers. This study allowed the identification of PVs specific to early-onset BC, which may be used as a foundation to develop regional expertise and diagnostic tools for early detection of BC in young Kazakh women.

## INTRODUCTION

In Kazakhstan, breast cancer (BC) is the most common malignancy in women. According to the latest national study on the epidemiology of BC in Kazakhstan, the number of reported cases increased by 14% between 2017 and 2021 [1]. By nationality, most BC patients with a confirmed primary diagnosis are Kazakh (48.1%), followed by Russian (33.1%) [1]. A detailed study within ethnic groups shows that BC accounts for 26.3% of all cancer cases and 8.7% of cancer-related deaths in Kazakh women [2]. At the same time, although the prevalence of BC in Kazakhstan shows a steady upward trend in the age range from 42.5 years to 62.5 years, the prevalence of BC in young women has also increased from 309 cases in 2017 to 330 cases in 2021 [1]. Thus, combined with a poorer prognosis, more aggressive histologic features, and more frequent recurrences [3, 4], BC in young Kazakh women in Kazakhstan is a growing threat. The problem is exacerbated by the fact that unlike an effective golden standard of mammography screening for women aged 40–70 years, screening programs for BC in young women are still underdeveloped in Kazakhstan. Targeted development of molecular genetic biomarkers for genetic predisposition to early BC in Kazakh women could provide a useful choice of efficient and noninvasive diagnostic methods.

Genetic factors significantly contribute to the risk of early-onset BC. In the United States, approximately 5 to 10% of BC cases are believed to have a hereditary risk [5]. However, these percentages are higher in a subset of women with early-onset BC, familial history, or triple-negative BC (TNBC), i.e., estrogen and progesterone receptors (ER and PR, respectively) and human epidermal growth factor receptor 2 (HER2) negative, based on immunohistochemistry (IHC) [6, 7]. Moreover, the cumulative risk of developing BC by age 50 in sisters and mothers of patients under 40 years old with BC was 6 and 3 times higher than the average population, respectively [7].

BC-associated variants have variable penetrance. Rare, highly penetrant genes that confer a >5-fold relative risk [8] tend to cluster in families and are associated with hereditary BC subtypes. Moderate-penetrance variants that confer two- to five-fold increased risks [8] also contribute to a minority of BC cases. According to epidemiological studies, 15–20% of familial BC cases and less than 5% of all BCs are caused by germline variants in the *BRCA1* and *BRCA2* genes [9]. Carriers of pathogenic or likely pathogenic variants (PVs) in these genes have a 60–85% risk of developing BC by the age of 70 [10]. Results from population studies have shown that PVs in *BRCA1* and *BRCA2* are responsible for the development of BC in only 5–10% of cases [11]. Thus, other high-to-moderate-penetrance variants might also contribute to the onset of BC in the remaining cases. These include the rare highly penetrant variants in the *CDH1, PTEN, STK11*, *TP53* genes, and the moderately penetrant variants in the *ATM, CHEK2, PALB2* genes, among others. Genetic variants in these genes are associated with a 2–5-fold increase in the risk of developing BC [12–14]. Currently, according to the guidelines of the National Comprehensive Cancer Network (NCCN), preventive diagnostic and therapeutic measures are recommended for carriers of pathogenic variants in the *ATM, BARD1, BRCA1/BRCA2, CDH1, CHEK2, NF1, PALB2, PTEN, RAD51C/RAD51D, STK11*, and *TP53* genes.

The existing differences in genetic structure and history between different populations require identifying and analyzing the spectrum and prevalence of PVs in genes involved in carcinogenesis in specific ethnic groups. To date, little data are available on the hereditary cancer risk among Kazakh women with BC. To characterize the inherited BC risk among Kazakh women with early onset BC, we used next-generation sequencing (NGS) to identify PVs involved in early-onset BC, and assessed their relationship with clinical and prognostic factors. Our study may reveal previously uncharacterized population-specific variants that may increase the risk of BC in the Kazakh population.

## RESULTS

### Clinical characteristics of the study population

Our study enrolled 224 unrelated Kazakh women diagnosed with early onset BC. The median age at diagnosis was 34.6 with a range from 19 to 40 years. Of these, 35 patients (15.6%) were diagnosed with BC under the age of 30 and 31 patients (13.8%) had family history of BC. Pathological examination showed predominantly invasive ductal carcinoma (90.6%) and moderately (64.7%) and poorly differentiated grade (30.4%) of the tumor. At the time of diagnosis, most patients (71.4%) had stage II of the disease and 14.6% had metastases in the axillary lymph node. The majority of cases (88.4%) had *high* (*≥14*%) *Ki-67* expression, indicating rapid proliferation of cancer cells [15]. Immunohistochemistry (IHC) revealed that most patients had the aggressive subtypes of BC: TNBC (32.6%) and Luminal B (Her2 negative) (27.3%). The detailed clinicopathological characteristics of patients are summarized in Table 1.

**Table 1:** Characteristics of the study cohort and comparison of carriers with non-carriers of a pathogenic variants

Breast cancer (BC)	All patients No (%)	BRCA1/2 carriers No (%)	Non-BRCA carriers No (%)	Non-carriers No (%)	*P*
**No of patients**	224	38 (17.0)	19 (8.5)	167 (74.6)	
**Age at diagnosis, years**					
Median Age (Min-Max)	34.6 (19–40)	34.9 (27–40)	34.7 (23–40)	34.7 (19–4)	0.110
Age 19–30	43 (19.20)	9 (23.7)	7 (36.8)	27 (16.2)	0.079
Age 31–40	181 (80.80)	29 (76.3)	12 (63.1)	140 (83.8)	
**Family history of cancer**					
No family history of cancer	178 (79.5)	26 (68.4)	19 (100.0)	133 (79.6)	**0.041**
Breast cancer	31 (13.8)	10 (26.3)	0 (0.0)	21 (12.6)	
Other cancers	15 (6.7)	2 (5.3)	0 (0.0)	13 (7.8)	
**Histotype**					
Invasive ductal carcinoma	203 (90.6)	35 (92.1)	14 (73.7)	154 (92.2)	**0.034**
Invasive lobular carcinoma	12 (5.4)	3 (7.9)	3 (15.8)	6 (3.6)	
Others	9 (4.0)	0 (0.0)	2 (9.1)	7 (4.2)	
**Grade**					
Well differentiated	8 (3.6)	1 (2.6)	0 (0.0)	7 (4.2)	0.821
Moderately differentiated	145 (64.7)	24 (63.2)	11 (57.9)	110 (65.9)	
Poorly differentiated	68 (30.4)	12 (31.6)	8 (42.1)	48 (28.7)	
Not applicable	3 (1.4)	1 (2.7)	0 (0.0)	2 (1.2)	
**Stage**					
*In situ*	2 (0.9)	0 (0.0)	0 (0.0)	2 (1.2)	0.09
I	15 (6.7)	2 (5.3)	1 (5.3)	12 (7.2)	
II	160 (71.4)	29 (76.3)	9 (47.4)	122 (73.1)	
III	38 (17.0)	6 (15.8)	6 (31.6)	26 (15.6)	
IV	9 (4.0)	1 (2.6)	3 (15.8)	5 (3.0)	
**Axillary lymph node metastases (stages II–III)**					
Yes	29 (14.6)	5 (14.3)	6 (40.0)	18 (12.2)	**0.023**
No	169 (85.4)	30 (85.7)	9 (60.0)	130 (87.8)	
**Ki-67**					
High (≥14)	198 (88.4)	34 (89.5)	18 (94.7)	146 (87.4)	0.872
Low (<14)	24 (10.7)	4 (10.5)	1 (5.3)	19 (11.4)	
Not applicable	2 (0.9)	0 (0.0)	0 (0.0)	2 (1.2)	
**Molecular subtype**					
Luminal A (ER+ and/or PR+ HER2− KI67−)	27 (12.1)	3 (7.9)	2 (10.5)	22 (13.2)	**0.005**
Luminal B− (ER+ and/or PR+ HER2− KI67+)	61 (27.3)	9 (23.7)	7 (36.8)	45 (27.0)	
Luminal B+ (ER+ and/or PR+ HER2+ KI67+)	38 (17.1)	6 (15.8)	1 (5.3)	31 (18.6)	
HER2 overexpression (ER− and/or PR− HER2+)	23 (10.3)	1 (2.6)	3 (15.8)	19 (11.4)	
TNBC (ER− and/or PR− HER2−)	73 (32.6)	19 (50.0)	6 (31.6)	48 (28.7)	
Not applicable	2 (0.9)	0 (0.0)	0 (0.0)	2 (1.2)	

Abbreviations: ER: estrogen receptor; PR: progesterone receptor; HER2: human epidermal growth factor receptor 2; TNBC: triple negative breast cancer. Statistically significant value (*p* < 0.05) is denoted in bold.

### Spectrum of germline PVs

**Figure 1:**
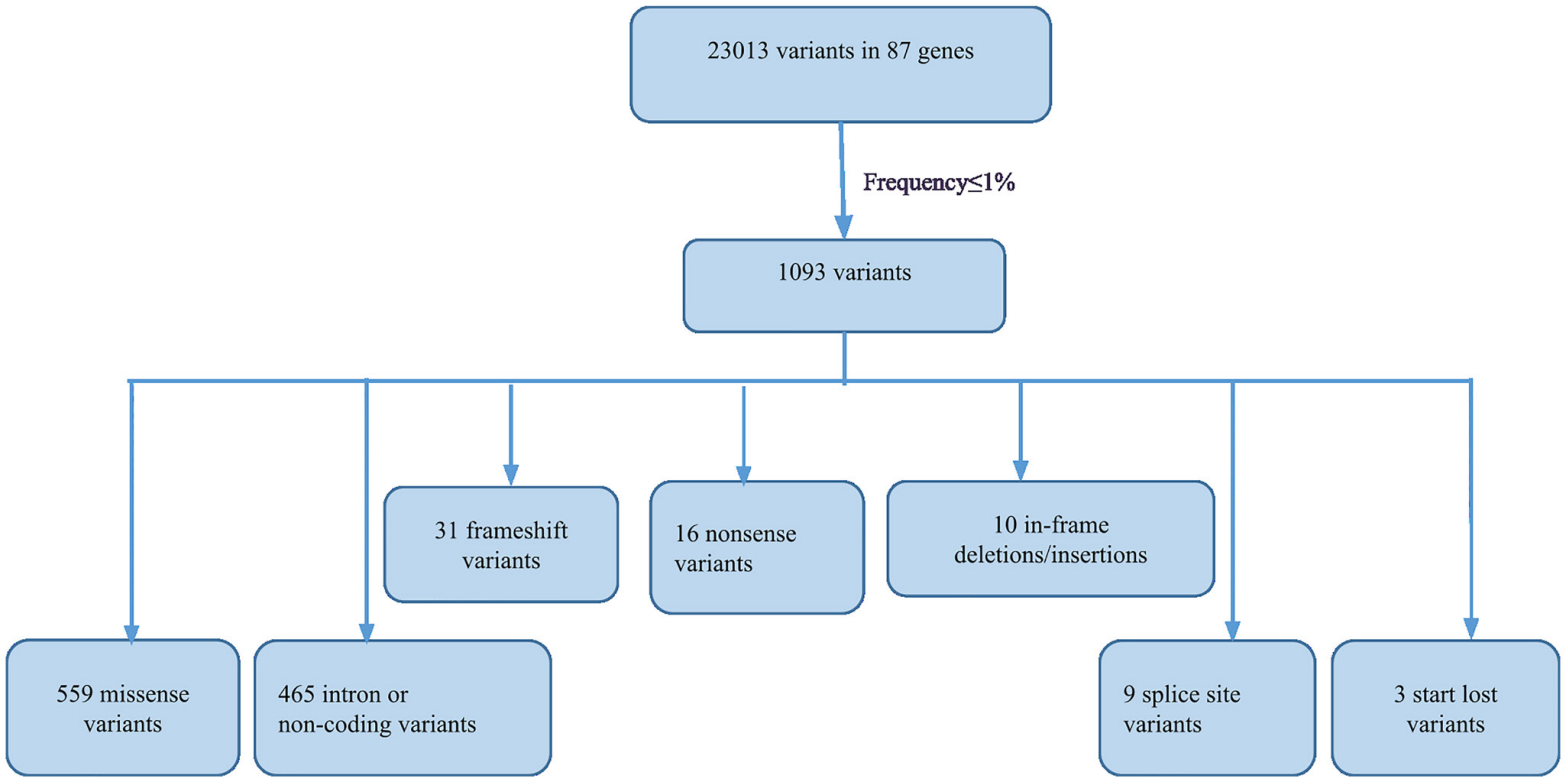
Flowchart illustrating the filtering and spectrum of rare variants detected by NGS.

A total of 23,013 unique variants in 87 genes were detected across 224 unrelated patients with early-onset BC. The identified variants were 7,720 missense, 12,727 synonymous, 1,230 3′-UTR, 668 5′-UTR, 31 frameshift, 16 nonsense, 10 in-frame, 9 splice site, 3 start-lost, and 599 intron or non-coding. Of these, 1,093 variants were found at frequency ≤1%. The spectrum of rare variants in the 224 patients is represented in Figure 1. To filter out rare polymorphisms (1093 variants, Figure 1), we eliminated variants that occur at a frequency of more than 1% in population databases 1000 Genomes database (1000G) [16], the Exome Sequencing Project database (ESP6500, https://esp.gs.washington.edu), and the Exome Aggregation Consortium database (ExAC).

In total, we identified 59 PVs (38 unique positions) located in the 13 genes, which occurred in 57 patients (25.4%) (Supplementary Table 1). The prevalence of PVs out of the total PVs identified in the 13 genes was as follows: *BRCA1* (37.3%), *BRCA2* (27.1%), *TP53* (8.5%), *CHEK2* (6.8%), *PALB2* (5.1%), *SDHB* (3.4%), *ATM* (1.7%), *BLM* (1.7%), *FANCM* (1.7%), *NBN* (1.7%), *PMS1* (1.7%), *PMS2* (1.7%), and *XPA* (1.7%). Thus, the most common pathogenic germline variants in 57 young patients with PVs were the *BRCA1* (39%, 22/57) gene, followed by the *BRCA2* (28%, 16/57), *TP53* (9%, 5/57), *CHEK2* (7%, 4/57), *PALB2* (5%, 3/57), and *SDHB* (4%, 2/57) genes. Other genes *ATM, BLM, FANCM, NBN, PMS1, PMS2*, and *XPA* were present in 14% of patients with PVs (2%, 11/57 for each gene).

Patients with multiple PVs were also found in our study. Specifically, two patients were carriers of two PVs in different cancer predisposition genes. One patient diagnosed with hormone-positive BC at the age of 31 years and a first-degree relative with BC (mother diagnosed with BC at the age of 45) had *BRCA2* c.9409dup in combination with *ATM* c.2465T>G. The other patient diagnosed with TNBC at the age of 39 was carrier of *BRCA1* c.2T>C and *BLM* c.320dup.

**Figure 2:**
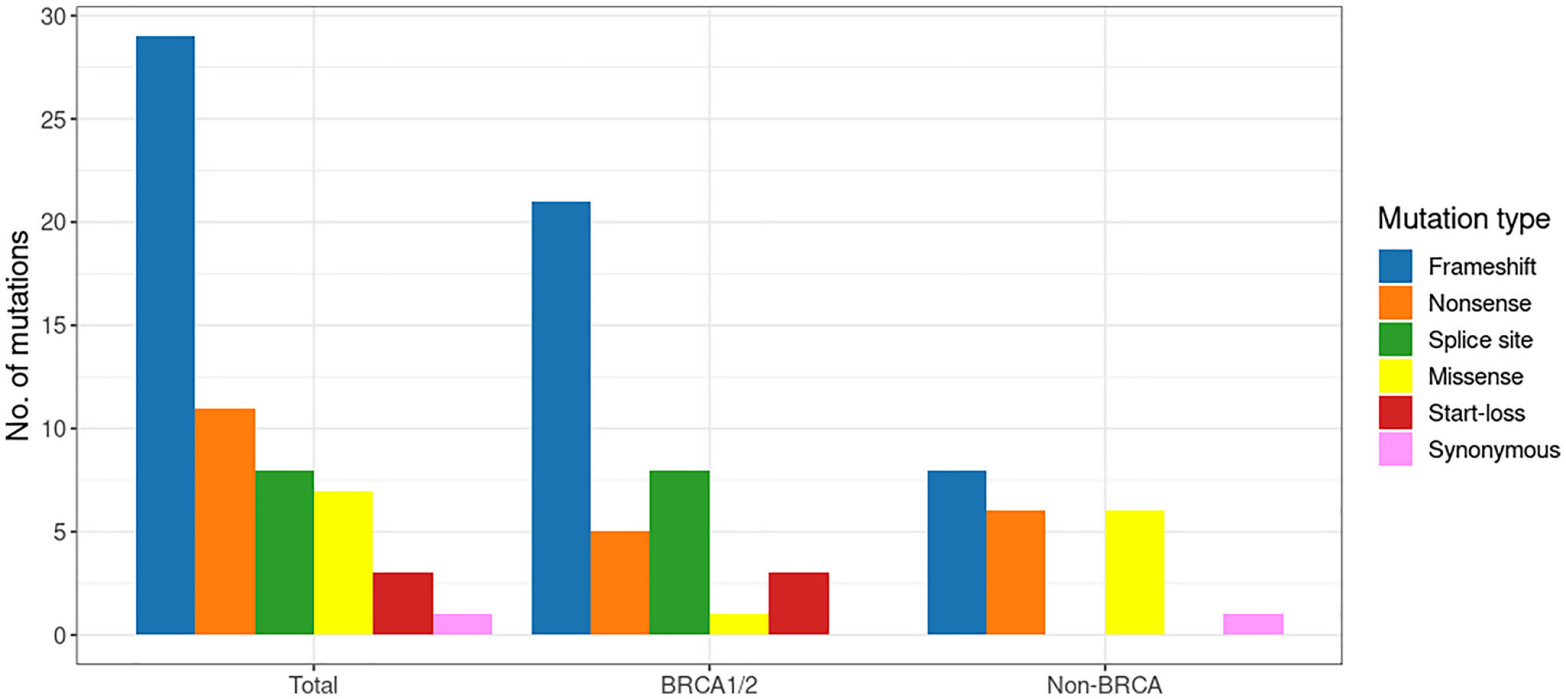
The spectrum of pathogenic variants in the early-onset breast cancer cohort.

The 59 PVs identified in the 57 patients included 29 frameshift, 11 nonsense, 8 splice site, 7 missense, 3 start-loss, and 1 synonymous. As shown in Figure 2, frameshift variants were the predominant type in both the *BRCA1/2* and non-*BRCA* groups. Spice site and start-loss variants were detected only in the *BRCA1/2* group. In the non-*BRCA* group, nonsense and missense variants were evenly distributed. All the detected PVs existed in a heterozygous state.

In addition to the 59 PVs, we also identified additional variants (5 in-frame, 2 nonsense, 1 frameshift and 1 splice site) in 16 patients that were not annotated neither as (likely) pathogenic nor as (likely) benign in LOVD, ClinVar, and ARUP’ databases. Thus, these were labeled as variants of unknown significance (VUS, Supplementary Table 2).

### Identification of recurrent PVs

**Figure 3:**
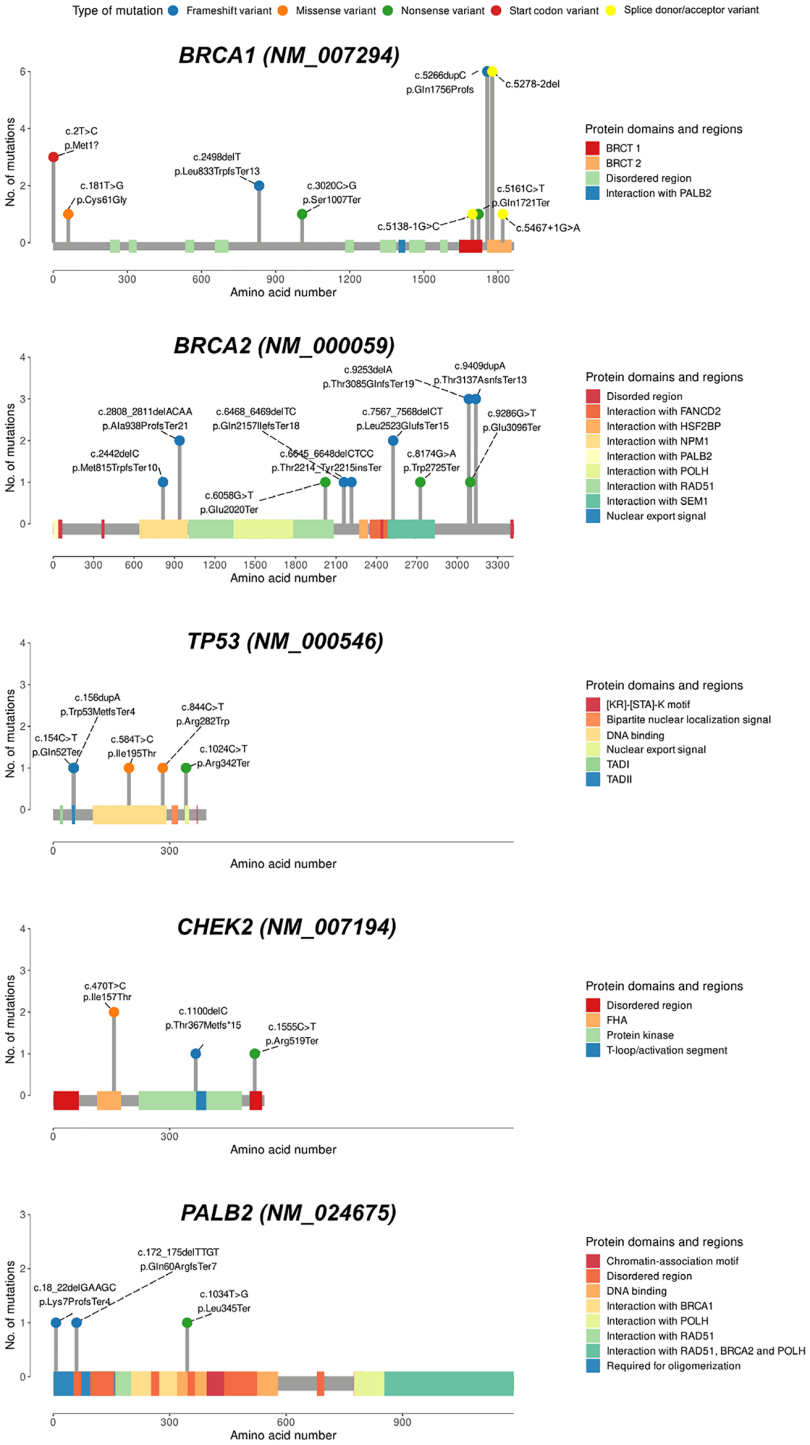
Lollipop charts of the genes with the highest number of variants in this study. The variant type is shown by colored balls. Protein domains are indicated as colored squares. BRCT1/2, BRCA1/2 C-terminus. Abbreviations: TAD: transactivation domain; FHA: forkhead-associated domain.

In total, 12 (31.6%) of the 38 unique PVs were seen recurrently. We identified five PVs that each occurred in ≥3 patients in our cohort (Figure 3). Of these, three variants were observed in the *BRCA1* gene, and two were detected in the *BRCA2* gene. The most common PVs were the frameshift insertion c.5266dup (also known as c.5382ins) and the splice acceptor site variant c.5278-2del (c.5341-2del) in *BRCA1* gene, each accounting for 2.7% (*n* = 6) of the cohort (Figure 3). Two frameshift variants (c.9409dup and c.9253del) in *BRCA2* gene and one nonsense variant in *BRCA1* (c.2T>C) were each observed in three patients. Five additional PVs (*BRCA2* c.2808_2811del, *BRCA2* c.7567_7568del, *BRCA1* c.2498del (novel), *CHEK2* c.470T>C, and *SDHB* c.725G>A) each occurred in two patients, whereas 25 PVs (65.8%) occurred in single patients.

### Identification of novel PVs

Overall, of the 59 PVs, we identified 6 novel PVs (*BRCA1* c.2498del; *PALB2* c.1034T>G, c.18_22del; *TP53* c.154C>T, *XPA* c.20del, *PMS1* c.1258del, Supplementary Table 1) that were not previously reported in any of the databases (LOVD, ClinVar, and ARUP). Among these, the novel frameshift deletion *BRCA1* c.2498del was found in two patients diagnosed with unilateral TNBC at young age (31 and 28 years) and with first-degree relatives with cancer.

### Rare missense variants and variants of unknown significance

A total of 559 rare missense variants (minor allele frequency (MAF) <1%) in the population frequency databases) were detected in this study. Except for 1.3% variants reported as pathogenic in both ClinVar and LOVD, most of the variants were classified as VUS. All of them were in a heterozygous state. In ClinVar, 46.3% of the rare missense variants were registered as VUS, 23.6% were not reported, and 28.8% were considered to be benign. When we evaluated the impact of rare missense variants on protein structure and function using in silico algorithms (SIFT and PolyPhen-2) [17, 18], 291 variants in 161 patients were identified as potentially deleterious and probably damaging according to at least one prediction tool (Supplementary Table 3), while 53 variants in 46 patients were reported to be deleterious by both prediction tools (Supplementary Table 4). Six patients were carriers of at least two VUS each, all found in non-*BRCA1/2* genes.

### Association between germline PVs and clinical factors

To analyze the impact of the identified PVs on the clinical and pathological characteristics of early onset BC patients, we divided the cohort into *BRCA1/2* carriers, non-*BRCA1/2* carriers, and non-carriers (of PVs) (Table 1). The number of patients with at least one first-degree relative with BC was significantly higher among *BRCA1/2* carriers (26.3%) compared to non-carriers (12.6%), while no family history of BC was reported among *BRCA1/2* non-carriers (*p* = 0.041). While then number of patients with invasive ductal carcinoma was high in all three groups, a significantly higher proportion was observed in *BRCA1/2* carriers (92.1%) and non-carriers (92.2%) compared to *BRCA1/2* non-carriers (73.7%, *p* = 0.034). A significantly higher proportion of non-*BRCA1/2* carriers (40.0%) had axillary lymph node metastases (stages II–III) compared with both *BRCA1/2* carriers (14.3%) and non-carriers (12.2%, *p* = 0.023). Molecular subtype assessment indicated that 50% of *BRCA1/2* carriers had TNBC, which was significantly higher than the frequency observed in non-*BRCA1/2* carriers (31.6%) and non-carriers (28.7%, *p* = 0.005).

### Biomarkers and prognostic factors

To investigate further the contribution of the identified PVs to clinical and pathological characteristics of patients with early onset BC, we divided the cohort into two groups (carriers and non-carriers), and tested their association with hormonal status (Table 2) and prognostic factors (Table 3). Since most of the PVs affect the *BRCA1* and *BRCA2* genes, we focused on associations with these genes.

**Table 2:** Association between BRCA1/2 pathogenic variants and hormonal status

Hormonal status	*BRCA1*			*BRCA2*		
	Carriers (%)	OR (95% CI)	*P*-value	Carriers (%)	OR (95% CI)	*P*-value
**ER status**						
Negative	17 (77.3)	1.00	0.004	3 (20)	1.00	0.08
Positive	5 (22.7)	0.22 (0.08–0.62)		12 (80)	2.96 (0.80–10.87)	
**PR status**						
Negative	17 (77.3)	1.00	0.006	5 (33.33)	1.00	0.40
Positive	5 (22.7)	0.23 (0.08–0.66)		10 (66.67)	1.59 (0.52–4.85)	
**Her2 status**						
Negative	21 (95.5)	1.00	0.036	10 (66.67)	1.00	0.77
Positive	1 (4.5)	0.11 (0.02–0.87)		5 (33.33)	1.20 (0.39–3.70)	
**TNBC**						
No	6 (27.3)	1.00	**0.0002**	12 (80)	1.00	0.56
Yes	16 (72.7)	**6.61 (2.44–17.91)**		3 (20)	0.62 (0.16–2.29)	

Abbreviations: OR: odds ratio; CI: confidence interval; ER: estrogen receptor; PR: progesterone receptor; HER2: human epidermal growth factor receptor 2; TNBC: triple negative breast cancer. Statistically significant value (*p* < 0.05) is denoted in bold.

**Table 3:** Association of *BRCA1/2* pathogenic variant carriers for prognostic factors compared to noncarriers

Clinical status	*BRCA1* Carriers (%)	Non- carriers	OR (95% CI)	*P*-value	*BRCA2* Carriers (%)	Non- carriers	OR (95% CI)	*P*-value
**Bone metastasis**								
Negative	21 (95.5)	153 (91.6)	1.00	0.54	14 (87.5)	153 (91.6)	1.00	0.58
Positive	1 (4.5)	14 (8.4)	0.52 (0.06–4.16)		2 (12.5)	14 (8.4)	1.56 (0.32–7.57)	
**Lymph node metastasis**								
No	19 (86.4)	147 (88.0)	1.00	0.82	13 (81.3)	147 (88.0)	1.00	0.44
Yes	3 (13.6)	20 (12.0)	1.16 (0.31–4.27)		3 (18.7)	20 (12.0)	1.70 (0.44–6.47)	
**Lung metastasis**								
No	20 (90.9)	152 (91.0)	1.00	0.99	14 (87.5)	152 (91.0)	1.00	0.64
Yes	2 (9.1)	15 (9.0)	1.01 (0.21–4.76)		2 (12.5)	15 (9.0)	1.45 (0.30–6.98)	
**Liver metastasis**								
No	21 (95.5)	159 (95.2)	1.00	0.96	14 (87.5)	159 (95.2)	1.00	0.21
Yes	1 (4.5)	8 (4.8)	0.95 (0.11–7.94)		2 (12.5)	8 (4.8)	2.84 (0.54–14.68)	
**CNS metastasisv**								
No	21 (95.5)	157 (94.0)	1.00	0.79	15 (93.7)	157 (94.0)	1.00	0.97
Yes	1 (4.5)	10 (6.0)	0.75 (0.09–6.14)		1 (6.3)	10 (6.0)	1.05 (0.12–8.74)	
**Family history of cancer**								
Negative	14 (63.6)	133 (79.6)	1.00		11 (73.3)	133 (79.6)	1.00	
Positive for BC	7 (31.8)	21 (12.6)	**3.17 (1.14–8.76)**	**0.03**	3 (20.0)	21 (12.6)	1.73 (0.44–6.71)	0.43
Positive for other cancers	1 (4.6)	13 (7.8)	0.73 (0.09-6.01)	0.77	1 (6.7)	13 (7.8)	0.93 (0.11–7.79)	0.95
**Histological grade**								
I–II	17 (77.3)	134 (81.3)	1.00	0.66	14 (82.4)	134 (81.3)	1.00	0.91
III–IV	5 (22.7)	31 (18.7)	1.27 (0.43–3.71)		3 (17.6)	31 (18.7)	0.93 (0.25–3.42)	

Abbreviations: OR: odds ratio; CI: confidence interval; BC: breast cancer; CNS: central nervous system. Statistically significant value (*p* < 0.05) is denoted in bold.

As shown in Table 2, the association with TNBC in *BRCA1* carriers was significant (OR = 6.61, 95% CI 2.44–17.91, *p* = 0.0002). We found a significant association between *BRCA1* carriers and a family history of BC (OR = 3.17, 95% CI 1.14–8.76, *p* = 0.03) whereas *BRCA2* carriers had worse prognostic outcomes of developing a metastatic phenotype but not statistically significant as shown in Table 3.

## DISCUSSION

### Pathogenic variants prevalence

In clinical practice, genetic testing for BC risks has been based chiefly on *BRCA1/2* gene analysis, despite new evidence suggesting the clinical significance of a broader number of cancer-related genes [19]. In this study, we used a targeted panel including a vast number of genes implicated in hereditary cancer syndromes and overall BC cancer predisposition.

To our knowledge, this is the first study using NGS technology to study the genetic predisposition to early-onset BC women from Kazakhstan and assess their impact on the patients’ clinical outcomes. Overall, 25.4% of Kazakh women were carriers of one or more PVs in the 94 cancer-associated genes analyzed. PVs in the 12 established BC predisposition genes (*ATM*, *BARD1*, *BRCA1, BRCA2, CDH1*, *CHEK2*, *NF1*, *PALB2*, *PTEN*, *RAD51C*, *RAD51D*, and *TP53*) were detected in 22.3% of our patient population, which is higher than that reported in the CARRIERS consortium that was a population-based study of hereditary BC genes in the United States (5.03%) [20]. When compared to other studies of early-onset BC, the proportion of PVs found in our study was lower than reported in the Greek population (31.5%), but similar to other studies in diverse populations [21, 22].

The genetic landscape of the identified PVs was dominated by the *BRCA1* gene, followed by the *BRCA2, TP53, CHEK2, PALB2*, and *SDHB* genes, and was consistent with the profile of early-onset BC and/or ovarian cancer in the Indian population and in BC patients in the Brazilian miscegenated population, where PVs in the *BRCA1* gene were also dominant, followed by the *BRCA2* and *TP53* genes [23, 24]. In contrast, the most common pathogenic germline variants in the Greek patients with early-onset BC were PVs in *CHEK2*, followed by *BRCA1/2* and *TP53* genes [21]. However, compared to the Kazakh women, no PVs were detected in the *PALB2, SDHB, ATM, BLM, FANCM, NBN, PMS1, PMS2*, and *XPA* genes among the 94 cancer-associated genes analyzed in the Greek and Indian populations, which may be due to the smaller samples. Interestingly, when the same panel of 94 cancer genes was examined in the group of TNBC *BRCA*-negative patients from Cyprus, germline PVs of the *PALB2* gene were found in older women but not in young [25]. At the same time, in a large-scale study of BC women aged 35–59 years, who were mainly of Northern and Western European descent, PVs in *CHEK2, ATM, PALB2,* and *PMS2* genes were the most frequent among the 25 cancer genes tested, after PVs in *BRCA1/2* [26].

### *BRCA1/2* pathogenic variants

Our finding suggests that germline PVs in the *BRCA1* and *BRCA2* genes are major contributors to early-onset BC in the young Kazakh female population. Among the 59 PVs detected in 57 patients (25.4% of patients), the majority of the PVs were found in *BRCA* genes (64.4% of total PVs or 67% of total PV patients), which reflects the high proportion of *BRCA1/2* variants in early-onset BC women reported in other ethnic groups [21, 26]. In our study, the five PVs in *BRCA1* identified in 15 patients may lead to defects in BRCT domains, while the five PVs in *BRCA2* occurring in 7 patients were found in functional domains that may affect interactions with *RAD51*, *NPM1*, and *SEM1* (Figure 3). It is known that PVs in these genes are most prevalent in patients with hereditary breast and/or ovarian cancer [21, 27–29]. However, some studies using a targeted sequencing approach have discovered a higher proportion (62–73%) of PVs in other cancer susceptibility genes other than *BRCA1/2* [23, 30], with nearly equal contribution to hereditary BC [26, 31].

### Non-*BRCA1/2* pathogenic variants

In our study, 35.6% of all PVs found in non-*BRCA* genes might also contribute to early onset BC. Overall, 21 PVs in 11 non-*BRCA* genes (*ATM*, *BLM*, *CHEK2*, *FANCM*, *NBN*, *PALB2*, *PMS1*, *PMS2*, *SDHB*, *TP53*, and *XPA*) were found in 21 patients. Two patients had both *BRCA* and non-*BRCA* mutated genes (*BRCA2* c.9409dup plus *ATM* c.2465T>G, and *BRCA1* c.2T>C plus *BLM* c.320dup), with both having family history of BC and poor clinical outcomes.

The most frequently mutated genes after *BRCA1/2* genes were *TP53* (5/224; 2.2%), followed by *CHEK2* (4/224; 1.8%) and *PALB2* (3/224; 1.3%) (Figure 3 and Supplementary Table 1). Among the studied cohort, we did not find any constitutional mosaicism in *TP53* variants. Sixteen out of 21 PVs detected in this study were protein-truncating (the majority being frameshift), potentially resulting in loss of protein function.

Even though the frequency and spectrum of PVs in Kazakh women resembled those reported in Guindalini R.S.C et al. [24], most BC-associated genes and variants differ between races and populations [32, 33]. Work is evolving to better understand the implications of multiple pathogenic variants in an individual in regard to how it impacts cancer phenotype, age of cancer onset and tumor biology [34]. It remains to be determined whether these variants modify the BC risk. There is still limited data on tumor pathogenesis among patients with concurrent PVs in cancer genes, and future studies may provide valuable insight in counseling and management of individuals with multiple germline PVs.

### Recurrent and founder pathogenic variants

A founder variant is a PV that is observed at high frequency in a given population due to the presence of the variant in a single ancestor or a small number of ancestors [35]. Previous studies have shown that different populations have founder genetic variants that affect BC risk [36–39].

We detected 12 (31.6%) PVs that occurred in multiple patients and affected 13.8% (31/224) of the patient cohort. The most prevalent PVs were *BRCA1* c.5278-2del and c.5266dup, each found in 2.7% of total case (Supplementary Table 1). The c.5278-2del found within the BRCT (*BRCA1* C-terminal) domain affects an acceptor splice site in intron 19 of the *BRCA1* gene, which disrupts RNA splicing by generating two aberrant transcripts (one lacking exon 21 and the other skipping 8 bp at the 5′-end of exon 21) that lead to the formation of a truncated protein [40]. The frameshift insertion c.5266dup in the last exon of the *BRCA1* mRNA transcript is predicted to escape nonsense-mediated decay and be expressed as a truncated protein (p.Gln1756Profs*74) that lacks the C-terminal BRCT domain.

There is no evidence to date about the founder effect of *BRCA1* c.5278-2del within different populations, whereas *BRCA1* c.5266dup was reported as one of the most frequent PVs in Eastern and Central Europe [41] and North Africa [42]. It is also discussed as a founder mutation for the development of BC in Uzbek, Ashkenazi Jewish, Tatar and Russian populations [43–46]. Previous studies of the *BRCA1* gene showed the absence of the c.5266dup PV in a cohort of 121 Kazakh patients with BC, mean age of 50.3 ± 11.5 years [47]. A previous study using Sanger sequencing to identify *BRCA* variants in sporadic BC women from Kazakhstan with an average age of 50–51 years did not reveal any recurrent PVs, including *BRCA1* c.5266dup and c.5278-2del [48]. It is worth noting the advantage of using NGS technology, which significantly expands the possibilities for detecting relevant variants, rather than the conventional genotyping methods. Since *BRCA1* c.5266dup and c.5278-2del were found at high frequency among patients under 40 years of age (mean age 34 years) in our cohort, we can assume that they are characteristic of the early development of BC, although future studies investigating the frequency distribution of these variants in patients subgroups by age are needed for confirmation. *BRCA1* c.5278-2del has been detected in one Italian patient with a personal and family history of BC and reported as a rare variant [49]. This variant is currently not reported in the 1000G, ESP6500 and ExAC databases. However, it was also previously identified in a colorectal cancer patient of Kazakh ethnicity [50]. Phelan et al. in their prospective cohort study showed four-fold increased risk of early-onset colorectal cancer in female *BRCA1* PVs carriers [51]. Our findings suggest that *BRCA1* c.5266dup and c.5278-2del may be founder PVs in the Kazakh population. This could have important implications for preventive screening and targeted treatments for BC in the Kazakhstan population.

Other common PVs identified in our study are *BRCA2* c.9253del (also known as c.9481del) and c.9409dup, and *BRCA1* c.2T>C, each accounting for 1.3% of the entire cohort. Both frameshift variants in exons 24 and 25 of the *BRCA2* gene trigger the nonsense-mediated decay by introducing a premature termination codon (p.Thr3085GlnfsTer19 and p.Thr3137AsnfsTer13) in the DNA-binding OB-fold C-terminal domain (OB3). *BRCA2* c.9409dup was reported in LOVD and ARUP, but not in ClinVar. This PV has also been identified at a frequency of 0.2% in a Chinese BC cohort, which is much lower than the frequency reported in our study [52]. *BRCA2* c.9253del was also detected at a low frequency in Korean BC patients [52–55]. A sequencing study on a cohort of 32 early-onset BC patients from multiple ethnic regions of China also found this variant in one woman of Kazakh ethnicity [56]. Thus, these results suggest that *BRCA2* c.9409dup and c.9253del could be specific to Asian populations. The *BRCA1* c.2T>C variant modifies the methionine residue at the initiation codon. This variant is not reported in any population frequency database, and no functional characterizations are available yet. However, in our study, c.2T>C occurred in three patients with TNBC, and a neighbor PV (c.1A>G) resulting in the loss of the initial 17 amino acids of *BRCA1* was also frequently identified in breast and ovarian cancer families [57, 58].

Overall, the profile of founder mutations for the Kazakh population appears to be represented by *BRCA1* c.5266dup, which is common in European, African, and Asian ethnic groups; *BRCA2* c.9409dup and c.9253del, which are specific to Asian populations; and *BRCA1* c.5278-2del and c.2T>C. The last two PVs could be ethnicity-specific founder mutations for Kazakhs compared to other Central Asian ethnicities, as they have not been discussed as founder PVs in these ethnicities. For example, the founder mutation profile in Tatar women with BC includes *BRCA1* c.5161C>T and c.300T>G and *BRCA2* c.468dup in addition to *BRCA1* c.5266dup [45]. The most used test for Russian BC and ovarian cancer patients includes *BRCA1* c.4153delA, c.185delAG and *BRCA2* c.6174delT in addition to *BRCA1* c.5266dup. At the same time, the inclusion of four additional recurrent *BRCA1* mutations c.C61G, c.2080delA, c.3819del5, and c.3875del4 in the assay led to a significant increase in the number of identified Russian mutation carriers [46, 59]. The spectrum of mutations identified in Uighur women with early-onset and sporadic BC was completely different from that of Kazakhs and includes *BRCA1* c.3180insA, c.3538insT, c.3694insAA, c.1963insT, c.3948G>C, c.3182A>C, and c.3538G>T [60].

Further investigation of the revealed founder variants in other cancers in Kazakhs could lead to overlapping results, as these PVs cause truncated or absent *BRCA1* and *BRCA2* proteins and have already been found in other malignancies where loss of function is a known disease mechanism. *BRCA1* c.5266dup has previously been found in prostate cancer [61] and pancreatic cancer [62], in addition to its known role in ovarian cancer [63–65]. c.5278-2del has been detected in colorectal cancer [50] and intrahepatic cholangiocarcinoma [66] while *BRCA2* c.9253del has also been reported for prostate cancer [67].

### Novel pathogenic variants

We also identified six novel PVs: *BRCA1* c.2498del; *PALB2* c.1034T>G, c.18_22del; *TP53* c.154C>T, *XPA* c.20del, and *PMS1* c.1258del. The regions affected by these variants appear to be critical for the function of the corresponding proteins. The *BRCA1* c.2498del variant results in a frameshift that alters the amino acid sequence of the protein starting at position 2498 and leads to a premature termination codon 13 amino acids downstream. The other frameshift variants *XPA* c.20del and PMS1 c.1258del generate a premature translational stop signal (p.Ala7ValfsTer8 and p.His420IlefsTer22, respectively) and result in a premature termination codon 8 and 22 amino acids downstream, respectively. The nonsense mutation *PALB2* c.1034T>G results from a substitution of T to G at nucleotide position 1034, changing the amino acid from a leucine to a stop codon in the protein, and the *TP53* c.154C>T nonsense variant produces a premature translation stop signal (p.Gln52Ter). All of these variants are expected to result in an absent or disrupted protein product. Because these variants have not previously been described in population frequency databases, we hypothesise that they may be specific to the Kazakh population. Our further studies will involve the clinical scrutiny of carriers with these PVs. It will help clarify the role of these pathogenic variants in the development and progression of breast cancer.

### Association between genetic profiles and clinicopathological features

Population-based studies indicate that BC patients with specific clinical features are more likely to have PVs in BC susceptibility genes [68]. In this study, *BRCA1* PVs carriers had a greater risk of having family history (statistically significant) whereas *BRCA2* carriers had worse prognostic outcomes of developing a metastatic phenotype (not statistically significant, Table 3). In our study, the proportion of *BRCA1/2* carriers with TNBC was 8.4%, which is within the range of those previously reported in other populations [69]. In line with the existing evidence [68], tumors from *BRCA1/2* carriers were more frequently ductal carcinomas, whereas non-*BRCA1/2* carriers variants were more likely to have lobular BC. Interestingly, family history of BC was not observed in non-*BRCA1/2* carriers, whereas 26.3% of *BRCA1/2* carriers and 12.6% of non-carriers had positive BC family history. The lack of family history of BC in non-*BRCA1/2* carriers could be explained by either the limited sample size of our study or the moderate/low penetrance of non-*BRCA1/2* genes.

Logistic regression analysis showed that the association with TNBC was almost 7-fold greater in *BRCA1* carriers, while *BRCA2* status was not significantly associated with TNBC. Patients with TNBC were highly enriched in *BRCA1* c.5278-2del, c.5382ins, c.2T>C, and c.2498del. It should be noted that *BRCA1* carriers were less likely to develop ER, PR, and Her2-positive BC than carriers of *BRCA2* variants (Table 2). These results are consistent with the findings from a previous study in Chinese women with BC, which also reported a strong association between *BRCA1* variants, but not *BRCA2* variants, and TNBC [33]. Unfortunately, we did not have sufficient clinical data or statistical power to perform a subpopulation analysis. The limited sample size would also prevent from obtaining reliable estimates from subpopulation analyses.

Although our efforts focused on BC family history, we were interested in how the identified strong PVs might be related to cancer development on the male side. In our study, this association was traced for cancer of the digestive system. The father of the patient with *BRCA2* c.6468_6469delTC/BIC: 6696delTC had laryngeal cancer, and the brother and father of the carrier of PV *BRCA1* c.2498delT had gastric and esophageal cancer. Numerous studies confirm that pathogenic BRCA germline variants play a predisposing role for gastrointestinal malignancies [70–73]. Interestingly, the analysis of personal data did not reveal a single case of cancer of the male reproductive organs among the patients’ relatives. However, cases of cancer of the male reproductive organs, generally prostate cancer, have been reported in the literature in carriers of germline mutations in the *BRCA1, TP53, NBN, PALB2, CHEK2*, and *ATM* genes [61, 74–78].

In conclusion, our study comprehensively analyzed the spectrum and prevalence of PVs with early-onset BC in unrelated young women of the Kazakh population. Using NGS-based multigene panel testing, we were able to identify recurrent, possible founder and novel PVs that were undetected in earlier studies due to a less comprehensive and sensitive methodology. Moreover, the identified PVs were differentially associated with biomarkers and prognostic factors. Since the average incidence in young women of all ethnicities in Kazakhstan is 329.6 cases per year [1], we have every reason to discuss the applicability of the results to the general population and the sufficient representativeness of our sample, consisting of 224 young Kazakh women.

We demonstrated the remarkable efficacy of an NGS-based panel to identify rare germline variants in early onset BC patients. These findings could contribute to the development of population-specific multigene panels for more rapid and cost-effective testing. Expanding routine genetic testing for hereditary BC from traditional *BRCA* testing to multigene panels could improve diagnostic yield, increase cancer prevention options for both identified carriers and their relatives, and reduce the likelihood that hereditary families with early onset BC will be overlooked [79]. Nevertheless, further studies are needed to validate the clinical utility of these panels. The significant gaps in knowledge regarding genotype–phenotype correlations, expressivity, and penetrance should be addressed [80]. To maximize clinical utility and minimize potentially harmful effects, the efficacy of each testing strategy should be evaluated very specifically, from test panels containing only founder PVs to test panels combining genes with high and moderate penetrance or even VUS [81]. A result with a high number of VUS is likely to be more expensive than useful and increases patient anxiety because the likelihood of detecting VUS increases when many genes are tested [82]. At last, the problem of disclosing genetic test results to young patients and family members and the problem of standardizing counseling and clinical management of carriers of gene variants should be reviewed [81]. In this way, the multi-gene panel test for hereditary BC will be adopted by health care providers as a screening tool once clear guidelines are available. Otherwise, inadequate implementation of genetic testing may result in high health care expenditures, wasted time, and other resources without a positive impact on health outcomes [82].

With this in mind, we will focus in the future on segregation analyzes of family members and functional analyzes to evaluate the inheritance pattern and pathogenicity of the identified recurrent and novel BC variants. Retrospective analyses of their possible association with progression-free, metastasis-free, and overall survival are also an exciting direction for future research. No less interesting would be the study of these variants regarding the chemosensitivity and efficacy of specific targeted therapies for their carriers.

## MATERIALS AND METHODS

### Study population and data collection

The study included 224 unrelated female patients from the Kazakh ethnic group diagnosed with BC at or under 40 years of age. All patients were treated and followed up at the Kazakh Institute of Oncology and Radiology (KazIOR, Almaty, Kazakhstan) from August 2017 to October 2019. The study protocol was approved by the Ethics Committee of the KazIOR and was conducted in accordance with the Declaration of Helsinki. All the participants signed the informed consent and donated 5 ml of blood for molecular-genetic analysis. The patients also gave consent for publishing the results of their molecular-genetic and clinical data anonymously under a specific ID code assigned for the study. Collected blood in EDTA tubes was transported to the Institute of Genetics and Physiology in a portable refrigerated container within several hours after the collection and frozen at −20°C for further molecular-genetic studies. Information about sociodemographic status, clinicopathological, and family history were obtained from all patients by the ordering physician. ER, PR, HER2, and antigen Ki-67 status were evaluated by IHC using standard procedures and manufacturer protocols. Histological grade was evaluated using the Nottingham grading system.

### DNA preparation

Rigid quality control procedures were carried out at several stages of the sequencing process throughout our work. The QIAamp DNA Blood Mini Kit (QIAGEN) was used to isolate DNA from peripheral blood samples while according to manufacturer recommendations. The quantification and quality evaluation of the isolated genomic DNA (gDNA) samples were then performed using spectrophotometry (Biophotometer plus, Eppendorf, Germany) and fluorometry (Qubit fluorometer, Invitrogen, USA). At least 50 ng of double-stranded nuclear DNA per sample were obtained. In order to prepare libraries, gDNA samples had to be broken up, and then tags had to be attached to the resulting pieces. The removal of undesirable components was ensured by subsequent purifying stages, which produced libraries of the highest caliber. Qubit was used to quantify these libraries, and an Agilent Bioanalyzer was used to determine their size distribution.

### NGS – library preparation and sequencing

According to the manufacturer’s recommended protocol, we performed NGS using the TruSight Rapid Capture Kit (Illumina) in combination with the TruSight Cancer Sequencing Panel (Illumina). TruSight Cancer Sequencing Panel is a specific enrichment system targeted to the whole coding regions (>1700 exons) and the flanking noncoding regions (on average 50 bp upstream and downstream each exon) of 94 high-risk genes associated with various types of cancer predisposition according to genome-wide association studies (GWAS). The panel covers a total of 255 kb of the human genome. The selection of this panel enables a comprehensive evaluation of putative cancer-related genetic variations.

For library preparation, 50 ng of each gDNA sample was fragmented by Nextera transposome and adapter sequences (tags) were attached to the ends of the fragments. The “tagmented” DNA fragments were cleaned up from the Nextera transposome taking into account the individual bar-code for each patient, and standard adapters required for cluster amplification were added. The “tagmented” DNA was amplified by PCR followed by purification of the amplified fragments. Purification of DNA library was carried out for removing unwanted amplification products using magnetic particles (Sample purification beads, SPB). The library was then qualitatively and quantitatively evaluated using a fluorometric method, such as Qubit Fluorometer (Invitrogen, USA) and by using an Agilent High Sensitivity DNA Kit (Agilent Technologies) on 2100 Bioanalyzer (Agilent Technologies, USA). Next, 500 ng of individual DNA libraries were combined into a single pool in batches of 24 samples followed by double hybridization to capture labeled probes specific to the targeted genomic regions of interest. Nonhybridized material was removed by washing. Before PCR amplification, the captured library was purified using SPB for removing nonhybridized material, and then the enriched library was amplified and re-purified with SPB. The full library was quantified using the Qubit Fluorometer (Invitrogen, USA) and library quality was assessed using Agilent High Sensitivity DNA Kit (Agilent Technologies, USA) on 2100 Bioanalyzer (Agilent Technologies, USA) as previously described. Finally, DNA libraries with 4 mM molarities were subjected to cluster generation using a standard flow cell.

Sequencing was carried out using the MiSeq platform and MiSeq Reagent Kit v3 (Illumina) in paired-end runs of 24 samples of each separate library as recommended by the manufacturer. To analyze 224 samples, 10 MiSeq Reagent Kits v3 were used, with 24 pooled samples each. The MiSeq Reagent Kit v3 provide the highest output among all MiSeq kits. It utilize the same pre-filled, ready-to-use reagent cartridges as the v1 and v2 kits, but incorporate enhanced chemistry to boost cluster density, extend read lengths up to 2 × 300 bp using the 600-cycle kit, and improve quality scores. We used PhiX library as a quality and calibration control for cluster generation, sequencing runs, alignment, and cross-talk matrix generation.

Mean target coverage was 170,7X, and a 95.4% of the targeted regions was covered by at least 20 reads. These values are sufficient to consider the sequencing results reliable and valid.

### NGS – bioinformatics data analysis

The bioinformatics data analysis was performed using two methodological approaches accompanying different algorithms. In the first approach, NGS data analysis employed MiSeq Reporter v.3.0 software (Illumina, USA) using the Burrows-Wheeler Aligner (BWA) algorithm. We mapped and aligned the raw demultiplexing reads and compared to the reference human genome (GRCH37/hg19). By utilizing the Genome Analysis Toolkit from (GATK, Broad Institute, Cambridge, USA), we searched and detected variants for specific regions of the genome.

In the second approach, we used various bioinformatics methods and software packages to optimize the workflow. By utilizing the Bowtie2 algorithm with a very sensitive local parameter, we mapped and aligned the sequence reads against the human reference genome version GRCH37/hg19. The quality of reading sequences was evaluated using FastQC and MultiQC. For both the tools default parameters were used. The SAM files were converted into BAM files by Picard Tools and SAMTools/BCF tools. Afterward, we used Java Runtime Environment and R Bioconductor software scripts to sort and index the mapped reads, remove intermediate files, merge the BAM files, and identify duplicates. For re-alignment, the mapped reads around the areas with insertions/deletions GATK were used, and its “Haplotype caller” strategy was used to filter and detect genomic variants. Ultimately, all variant call format (VCF) files obtained from GATK contained only alternative variants imported into the Variant Studio 3.0 software (Illumina, USA). In our study, we have only considered the variants identified by both approaches to increase the robustness of the analysis.

### Annotation, interpretation, and classification of variants

The genetic variants were annotated and interpreted by using the Variant Studio 3.0 software (Illumina, USA). The acceptance threshold value was selected in terms of a Q-score of 30, corresponding to an error rate of 0.1%. The poor-quality nucleotide variants were excluded by utilizing the following filtering parameters: (i) a read depth of greater than 50×, (ii) alternative read depth of greater than 20×, and (iii) quality value of greater than 100. In this study, only variants that matched the filtering parameters were selected.

The annotation of genetic variants was performed in accordance with the nomenclature of the Human Genome Variation Society (HGVS) [83], and the interpretation was accomplished using the Single Nucleotide Polymorphism Database (dbSNP, http://www.ncbi.nlm.nih.gov/projects/SNP/), ClinVar database (https://www.ncbi.nlm.nih.gov/clinvar/), LOVD, (https://www.lovd.nl/), BRCA Exchange (https://brcaexchange.org/), and ARUP’ (https://arup.utah.edu/database/). The Integrative Genomics Viewer (IGV) [84–85] was employed to visualize both the quality and variance of the BAM and VCF files. Variants were considered rare if the minor allele frequency (MAF) was ≤1% according to 1000G, ESP6500, and ExAC.

Following the guidelines of the American College of Medical Genetics and Genomics (ACMG) [86], the identified variants were categorized as pathogenic, likely pathogenic, VUS, benign, and likely benign. According to the results of functional studies, pathogenic variants included those resulting in a premature stop codon (frameshift and nonsense), variants with uncorrected splicing, and variants affecting protein function. Novel frameshift and nonsense variants leading to protein truncation and variants without well-established functional effects were classified as likely pathogenic.

The ClinVar database was also used to classify variants based on previously established pathogenic or benign effects. Synonymous and intronic variants not affecting splicing were regarded as benign/probably benign. The remaining variants that did not exhibit functional effect and did not satisfy the classification criteria as pathogenic/likely pathogenic or benign/likely benign, or with inconclusive evidence of their benign and pathogenic nature, were defined as VUS.

*In silico,* bioinformatics tools such as SIFT [17] and PolyPhen-2 [18] were used to predict potential pathogenic effects of missense variants on protein structure and function. We considered variants with a SIFT score of less than 0.05 and a PolyPhen-2 score of more than 0.95 strongly suspected of being deleterious [87].

### Statistical analysis

We analyzed the distribution of patients’ characteristics, such as demographics, clinical-pathological characteristics, and personal and family history, between patient groups. The descriptive statistics were summarized as frequency distributions for categorical variables and as means for continuous variables. Kruskal-Wallis test and Fisher’s exact test were used to compare continuous and categorical variables between groups, respectively. The odds ratio (OR) and 95% confidence interval (CI) were calculated through logistical regression. Results were considered statistically significant at a *p*-value < 0.05. All statistical tests are two-sided at the 5% level of significance. The statistical data were processed and analyzed using the R v4.1.2 software.

## SUPPLEMENTARY MATERIALS











## Abbreviations

BC: breast cancer
TNBC: triple-negative breast cancer
NGS: next-generation sequencing
PV: pathogenic variant
VUS: variants of unknown significance
OR: odds ratio
CI: confidence interval
ER: estrogen receptor
PR: progesterone receptor
HER2: human epidermal growth factor receptor 2
NCCN: National Comprehensive Cancer Network
ACMG: American College of Medical Genetics and Genomics
HGVS: Human Genome Variation Society
LOVD: Leiden Open Variation Database
ARUP’: Associated Regional and University Pathologists
MAF: minor allele frequency
BWA: Burrows-Wheeler Aligner
ExAC: Exome Aggregation Consortium database
ESP6500: Exome Sequencing Project database
1000G: 1000 Genomes database
GATK: Genome Analysis Toolkit
BAM: Binary Alignment Map
SAM: Sequence Alignment Map
gDNA: genomic DNA
GWAS: genome-wide association studies
IHC: immunohistochemistry
CNS: central nervous system
EDTA: ethylenediaminetetraacetic acid
PCR: polymerase chain reaction
