# Supplementary material for: Determination of genetic predisposition to early breast cancer in women of Kazakh ethnicity
Source: Oncotarget. 2023 Oct 4;14:860–77. doi: 10.18632/oncotarget.28518 (PMC10549772; doi:10.18632/oncotarget.28518)
Supplement: Supplementary file 1 [file oncotarget-14-28518-s001.pdf]

Determination of genetic predisposition to early breast cancer in women of Kazakh ethnicity

SUPPLEMENTARY MATERIALS

Supplementary Table 1: Characteristic of pathogenic and likely pathogenic variants identified by Trusight cancer sequencing panel in 57 early-onset BC patients. See Supplementary Table 1.

Supplementary Table 2: Characteristics of variants whose clinical significance is not registered as pathogenic by LOVD and ClinVar databases in the cohort of patients with early-onset breast cancer. See Supplementary Table 2.

Supplementary Table 3: List of rare missense variants identified in the cohort of patients with early-onset breast cancer. See Supplementary Table 3.

Supplementary Table 4: Rare missense variants strongly suspected of being deleterious. See Supplementary Table 4.
