# Supplementary material for: Determination of genetic predisposition to early breast cancer in women of Kazakh ethnicity
Source: Oncotarget. 2023 Oct 4;14:860–77. doi: 10.18632/oncotarget.28518 (PMC10549772; doi:10.18632/oncotarget.28518)
Supplement: Supplementary file 2 [file oncotarget-14-28518-s002.docx]

**Supplementary Table 1: Characteristic of pathogenic and likely pathogenic variants identified by Trusight cancer sequencing panel in 57 early-onset BC patients**

| № | Patient ID | Gene | Chr | Genotype | Type of mutation | HGVSc | HGVSp | dbSNP ID | Population frequency | | | Database |
| --- | --- | --- | --- | --- | --- | --- | --- | --- | --- | --- | --- | --- |
|  |  |  |  |  |  |  |  |  | 1000G | Esp6500 | ExAC |  |
| 1 | BR39 | *PMS2* | 7 | Het | Synonymous variant | c.825A>G |  |  | NA | NA | NA | LOVD - pathogenic;  ClinVar- likely pathogenic​ |
| 2 | BR42 | *BRCA2* | 13 | Het | Frameshift variant | c.6468_6469delTC/BIC: 6696delTC | p.Gln2157IlefsTer18 | rs80359596 | NA | NA | NA | LOVD/ClinVar-pathogenic​ |
| 3 | BR45 | *BRCA2* | 13 | Het | Nonsense variant | c.8174G>A | p.Trp2725Ter | rs730881581 | NA | NA | NA | LOVD/ClinVar-pathogenic​ |
| 4 | BR49 | *BRCA1* | 17 | Het | Missense variant | c.181T>G/n.300T>G | p.Cys61Gly | rs28897672 | NA | NA | 0,01 | LOVD/ClinVar-pathogenic​ |
| 5 | BRC60 | *BRCA1* | 17 | Het | Frameshift variant | c.5266dupC/BIC:5382insC | p.Gln1756Profs | rs80357906 | NA | NA | 0,02 | LOVD/ClinVar-pathogenic​ |
| 6 | BRC65 | *NBN* | 8 | Het | Frameshift variant | c.657_661delACAAA/657del5 | p.Lys219AsnfsTer16 | rs587776650 | NA | 1,45 | 0,02 | LOVD/ClinVar-pathogenic​ |
| 7 | BRC69 | *TP53* | 17 | Het | Missense variant | c.844C>T | p.Arg282Trp | rs28934574 | NA | 0,02 | NA | LOVD/ClinVar-pathogenic​ |
| 8 | BRC73 | *TP53* | 17 | Het | Nonsense variant | c.1024C>T | p.Arg342Ter | rs730882029 | NA | NA | NA | LOVD/ClinVar-pathogenic |
| 9 | BRC80 | *BRCA2* | 13 | Het | Frameshift variant | c.2808_2811delACAA/BIC:3036delACAA | p.Ala938ProfsTer21 | rs80359351 | NA | 0,03 | NA | LOVD/ClinVar-pathogenic |
| 10 | BRC89 | *BRCA2* | 13 | Het | Frameshift variant | c.9253delA | p.Thr3085GlnfsTer19 | rs397508041 | NA | NA | NA | LOVD/ClinVar-pathogenic |
| 11 | BRC90 | *BRCA1* | 17 | Het | Frameshift variant | c.5266dupC/BIC:5382insC | p.Gln1756Profs | rs80357906 | NA | NA | 0,02 | LOVD/ClinVar-pathogenic |
| 12 | BRC99 | *BRCA1* | 17 | Het | Frameshift variant | c.5266dupC/BIC:5382insC | p.Gln1756Profs | rs80357906 | NA | NA | 0,02 | LOVD/ClinVar-pathogenic |
| 13 | BRC109 | *BRCA2* | 13 | Het | Frameshift variant | c.7567_7568delCT/BIC:7795delCT and also c.7563_7564CT | p.Leu2523GlufsTer15 | rs80359664 | NA | NA | NA | LOVD/ClinVar-pathogenic |
| 14 | BRC110 | *BRCA1* | 17 | Het | Frameshift variant | c.5266dupC/BIC:5382insC | p.Gln1756Profs | rs80357906 | NA | NA | 0,02 | LOVD/ClinVar-pathogenic |
| 15 | BRC115 | *BRCA1* | 17 | Het | Frameshift variant | c.5266dupC/BIC:5382insC | p.Gln1756Profs | rs80357906 | NA | NA | 0,02 | LOVD/ClinVar-pathogenic |
| 16 | BRC121 | *PALB2* | 16 | Het | Frameshift variant | c.172_175delTTGT | p.Gln60ArgfsTer7 | rs180177143 | NA | NA | NA | LOVD/ClinVar-pathogenic |
| 17 | BRC125 | *BRCA1* | 17 | Het | Nonsense variant | c.3020C>G | p.Ser1007Ter | rs80357168 | NA | NA | NA | LOVD/ClinVar-pathogenic |
| 18 | BRC128 | *BRCA1* | 17 | Het | Splice donor variant | c.5467+1G>A/BIC:5586+1G>A/IVS23+1G>A/c.5530+1G>A |  | rs80358145 | NA | NA | NA | LOVD/ClinVar-pathogenic |
| 19 | BRC130 | *TP53* | 17 | Het | Missense variant | c.584T>C | p.Ile195Thr | rs760043106 | NA | NA | NA | ClinVar-pathogenic; LOVD- NA |
| 20 | BRC149 | *BRCA1* | 17 | Het | Splice acceptor variant | c.5138-1G>C/BIC: 5194-1G>C/IVS17-1G>C |  | rs1800747 | NA | NA | NA | LOVD/ClinVar-pathogenic |
| 21 | BRC154 | *SDHB* | 1 | Het | Missense variant | c.725G>A | p.Arg242His | rs74315368 | NA | NA | NA | LOVD/ClinVar-pathogenic |
| 22 | BR36 | *PALB2* | 16 | Het | Nonsense variant | c.1034T>G | p.Leu345Ter |  | NA | NA | NA | **Novel** |
| 23 | BR38 | *PALB2* | 16 | Het | Frameshift variant | c.18_22delGAAGC | p.Lys7ProfsTer4 |  | NA | NA | NA | **Novel** |
| 24 | BR41 | *BRCA1* | 17 | Het | Splice acceptor variant | c.5278-2del/c.5341-2delA/IVS20-2delA |  |  | NA | NA | NA | LOVD/ClinVar-pathogenic​ |
| 25 | BrC7 | *TP53* | 17 | Het | Frameshift variant | c.156dupA/ c.39dupА (p.Trp14Metfs) | p.Trp53MetfsTer4 | rs1555526748 | NA | NA | NA | ClinVar-pathogenic; LOVD- NA  COSMIC |
| 26 | BRC52 | *BRCA1* | 17 | Het | Splice acceptor variant | c.5278-2del/ c.5341-2delA/ IVS20-2delA |  |  | NA | NA | NA | LOVD/ClinVar-pathogenic​ |
| 27 | BRC70 | *BRCA1* | 17 | Het | Frameshift variant | c.2498delT | p.Leu833TrpfsTer13 |  | NA | NA | NA | **Novel** |
| 28 | BRC81 | *BRCA1* | 17 | Het | Splice acceptor variant | c.5278-2del/ c.5341-2delA/ IVS20-2delA |  |  | NA | NA | NA | LOVD/ClinVar-pathogenic​ |
| 29 | BRC82 | *XPA* | 9 | Het | Frameshift variant | c.20delC | p.Ala7ValfsTer8 |  | NA | NA | NA | **Novel** |
| 30 | BRC96 | *FANCM* | 14 | Het | Nonsense variant | c.4270C>T | p.Arg1424Ter | rs751954386 | NA | NA | NA | ClinVar-pathogenic; LOVD- NA |
| 31 | BRC97 | *BRCA2* | 13 | Het | Frameshift variant | c.2442delC | p.Met815TrpfsTer10 | rs397507627 | NA | NA | NA | LOVD/ClinVar-pathogenic |
| 32 | BRC106 | *PMS1* | 2 | Het | Frameshift variant | c.1258delC | p.His420IlefsTer22 |  | NA | NA | NA | **Novel** |
| 33 | BRC113 | *BRCA2* | 13 | Het | Nonsense variant | c.6058G>T | p.Glu2020Ter | rs80358842 | NA | NA | NA | LOVD/ClinVar-pathogenic |
| 34 | BRC117 | *BRCA1* | 17 | Het | Nonsense variant | c.5161C>T/c.5224C>T | p.Gln1721Ter |  | NA | NA | NA | LOVD/ClinVar-pathogenic |
| 35 | BRC140 | *BRCA1* | 17 | Het | Splice acceptor variant | c.5278-2del/ c.5341-2delA/ IVS20-2delA |  |  | NA | NA | NA | LOVD/ClinVar-pathogenic​ |
| 36 | BRC158 | *BRCA1* | 17 | Het | Start lost variant | c.2T>C | p.Met1? | rs80357111 | NA | NA | NA | LOVD/ClinVar-pathogenic |
| 37 | BRC160 | *TP53* | 17 | Het | Nonsense variant | c.154C>T | p.Gln52Ter |  | NA | NA | NA | **Novel** |
| 38 | BRC162 | *BRCA2* | 13 | Het | Frameshift variant | c.9253delA/c.9481delA) | p.Thr3085GlnfsTer19 | rs397508041 | NA | NA | NA | LOVD/ClinVar-pathogenic |
| 39 | BRC163 | *ATM* | 11 | Het | Nonsense variant | c.2465T>G | p.Leu822Ter |  | NA | NA | NA | ClinVar-pathogenic; LOVD- NA |
|  |  | *BRCA2* | 13 | Het | Frameshift variant | c.9409dupA | p.Thr3137AsnfsTer13 |  | NA | NA | NA | ARUP'-pathogenic; LOVD-pathogenic; ClinVar-NA |
| 40 | BRC167 | *BRCA2* | 13 | Het | Frameshift variant | c.7567_7568delCT/ BIC: c.7795delCT/c.7563_7564CT | p.Leu2523GlufsTer15 | rs80359664 | NA | NA | NA | LOVD/ClinVar-pathogenic |
| 41 | BRC179 | *CHEK2* | 22 | Het | Frameshift variant | c.1100delC/c.1229delC | p.Thr367Metfs*15 | rs555607708 | 0,1 | 0,12 | 0,18 | LOVD/ClinVar-conflicting interpretations of pathogenicity​, likely pathogenic​ |
| 42 | BRC185 | *BRCA2* | 13 | Het | Frameshift variant | c.6645_6648delCTCC | p.Thr2214_Tyr2215insTer | rs886040672 | NA | NA | NA | LOVD/ClinVar-pathogenic​ |
| 43 | BRC188 | *BRCA1* | 17 | Het | Splice acceptor variant | c.5278-2del/ c.5341-2delA/ IVS20-2delA |  |  | NA | NA | NA | LOVD/ClinVar-pathogenic​ |
| 44 | BRC191 | *BRCA1* | 17 | Het | Frameshift variant | c.2498delT | p.Leu833TrpfsTer13 |  | NA | NA | NA | **Novel** |
| 45 | BRC205 | *BRCA2* | 13 | Het | Frameshift variant | c.9253delA | p.Thr3085GlnfsTer19 | rs397508041 | NA | NA | NA | LOVD/ClinVar-pathogenic |
| 46 | BRC206 | *BRCA1* | 17 | Het | Splice acceptor variant | c.5278-2del/ c.5341-2delA/ IVS20-2delA |  |  | NA | NA | NA | LOVD/ClinVar-pathogenic​ |
| 47 | BRC217 | *CHEK2* | 22 | Het | Nonsense variant | c.1555C>T/c.1684C>T | p.Arg519Ter | rs200432447 | NA | NA | NA | LOVD/ClinVar-pathogenic |
| 48 | BRC219 | *BRCA2* | 13 | Het | Frameshift variant | c.2808_2811delACAA/BIC:3036delACAA | p.Ala938ProfsTer21 | rs80359351 | NA | 0,03 | NA | LOVD/ClinVar-pathogenic |
| 49 | BRC222 | *BRCA2* | 13 | Het | Nonsense variant | c.9286G>T | p.Glu3096Ter | rs80359199 | NA | NA | NA | LOVD/ClinVar-pathogenic |
| 50 | BRC230 | *BRCA1* | 17 | Het | Frameshift variant | c.5266dupC/BIC:5382insC | p.Gln1756Profs | rs80357906 | NA | NA | 0,02 | LOVD/ClinVar-pathogenic |
| 51 | BRC234 | *BLM* | 15 | Het | Frameshift variant | c.320dupT | p.Leu107PhefsTer36 | rs781221411 | NA | NA | NA | LOVD/ClinVar-pathogenic |
|  |  | *BRCA1* | 17 | Het | Start lost variant | c.2T>C | p.Met1Thr | rs80357111 | NA | NA | NA | LOVD/ClinVar-pathogenic |
| 52 | BRC236 | *BRCA1* | 17 | Het | Start lost variant | c.2T>C | p.Met1Thr | rs80357111 | NA | NA | NA | LOVD/ClinVar-pathogenic |
| 53 | BRC159 | *CHEK2* | 22 | Het | Missense variant | c.470T>C | p.Ile157Thr | rs17879961 | 0,1 | 0,16 | 0,41 | LOVD/ClinVar-pathogenic |
| 54 | BRC187 | *SDHB* | 1 | Het | Missense variant | c.725G>A | p.Arg242His | rs74315368 | NA | NA | NA | LOVD/ClinVar-pathogenic |
| 55 | BRC223 | *CHEK2* | 22 | Het | Missense variant | c.470T>C | p.Ile157Thr | rs17879961 | 0,1 | 0,16 | 0,41 | LOVD/ClinVar-pathogenic |
| 56 | BRC58 | *BRCA2* | 13 | Het | Frameshift variant | c.9409dupA | p.Thr3137AsnfsTer13 |  | NA | NA | NA | ARUP'-pathogenic; LOVD-pathogenic; ClinVar-NA |
| 57 | BRC229 | *BRCA2* | 13 | Het | Frameshift variant | c.9409dupA | p.Thr3137AsnfsTer13 |  | NA | NA | NA | ARUP'-pathogenic; LOVD-pathogenic; ClinVar-NA |

Abbreviations: NA, not available; Het, heterozygote.
