# Supplementary material for: Determination of genetic predisposition to early breast cancer in women of Kazakh ethnicity
Source: Oncotarget. 2023 Oct 4;14:860–77. doi: 10.18632/oncotarget.28518 (PMC10549772; doi:10.18632/oncotarget.28518)
Supplement: Supplementary file 3 [file oncotarget-14-28518-s003.docx]

**Supplementary Table 2: Characteristics of variants whose clinical significance is not registered as pathogenic by LOVD and ClinVar databases in the cohort of patients with early-onset breast cancer**

| **№** | **Patient ID** | **Gene** | **Chr** | **Genotype** | **Type of mutation** | **HGVSc** | **HGVSp** | **dbSNP ID** | **Population frequency** | | | **Database** |
| --- | --- | --- | --- | --- | --- | --- | --- | --- | --- | --- | --- | --- |
|  |  |  |  |  |  |  |  |  | **1000G** | **EVS** | **ExAC** |  |
| 1 | BrC7 | *WRN* | 8 | het | Nonsense variant | c.4216C>T | p.Arg1406Ter | rs11574410 | 0,54 | 0,08 | 0,34 | LOVD, ClinVar- likely benign |
| 2 | BRC88 | *WRN* | 8 | het | Nonsense variant | c.4216C>T | p.Arg1406Ter | rs11574410 | 0,54 | 0,08 | 0,34 | LOVD, ClinVar- likely benign |
| 3 | BRC106 | *MSH6* | 2 | het | Frameshift variant | c.4068_4071dupGATT | p.Lys1358AspfsTer2 | rs267608142 | 0 | 0 | 0,23 | LOVD, ClinVar- likely benign, VUS |
| 4 | BRC112 | *BRCA2* | 13 | het | Nonsense variant | c.9976A>T | p.Lys3326Ter | rs11571833 | 0,44 | 0,65 | 0,7 | LOVD, ClinVar- likely benign, VUS |
| 5 | BRC150 | *BRCA2* | 13 | het | Nonsense variant | c.9976A>T | p.Lys3326Ter | rs11571833 | 0,44 | 0,65 | 0,7 | LOVD, ClinVar- likely benign, VUS |
| 6 | BRC176 | *BRCA2* | 13 | het | Nonsense variant | c.9976A>T | p.Lys3326Ter | rs11571833 | 0,44 | 0,65 | 0,7 | LOVD, ClinVar- likely benign, VUS |
| 7 | BRC189 | *MSH6* | 2 | het | Frameshift variant | c.4068_4071dupGATT | p.Lys1358AspfsTer2 | rs267608142 | 0 | 0 | 0,23 | LOVD, ClinVar- likely benign, VUS |
| 8 | BRC228 | *MSH6* | 2 | het | Frameshift variant | c.4068_4071dupGATT | p.Lys1358AspfsTer2 | rs267608142 | 0 | 0 | 0,23 | LOVD, ClinVar- likely benign, VUS |
| 9 | BrC7 | *EZH2* | 7 | het | In-frame insertion | c.566_568dupATG | p.Asp189dup | rs751123994 | 0 | 0 | 0 | Not reported (Novel) |
| 10 | BRC101 | *BRCA2* | 13 | het | In-frame deletion | c.4146_4148delAGA | p.Glu1382del | rs746421662 | 0 | 0,07 | 0,01 | ClinVar- VUS |
| 11 | BRC114_S24 | *BRCA2* | 13 | het | In-frame deletion | c.4146_4148delAGA | p.Glu1382del | rs746421662 | 0 | 0,07 | 0,01 | ClinVar- VUS |
| 12 | BRC158 | *RET* | 10 | het | In-frame insertion | c.56_58dupTGC | p.Leu19dup |  | 0 | 0 | 0 | ClinVar- VUS |
| 13 | BRC183 | *TSC2* | 16 | het | In-frame deletion | c.4527_4529delCTT | p.Phe1510del | rs137854239 | 0 | 0,44 | 0,53 | ClinVar- VUS |
| 14 | BRC187 | *BRCA2* | 13 | het | In-frame deletion | c.4146_4148delAGA | p.Glu1382del | rs746421662 | 0 | 0,07 | 0,01 | ClinVar- VUS |
| 15 | BRC191 | *TSC2* | 16 | het | In-frame deletion | c.4527_4529delCTT | p.Phe1510del | rs137854239 | 0 | 0,44 | 0,53 | LOVD, ClinVar- likely benign |
| 16 | BRC191 | *ERCC2* | 19 | het | In-frame deletion | c.1244_1246delCCA | p.Thr415del | rs778831415 | 0 | 0 | 0 | Not reported (Novel) |
| 17 | BRC203 | *RET* | 10 | het | In-frame insertion | c.56_58dupTGC | p.Leu19dup |  | 0 | 0 | 0 | ClinVar-VUS |
| 18 | BRC228 | *TSC2* | 16 | het | In-frame deletion | c.4527_4529delCTT | p.Phe1510del | rs137854239 | 0 | 0,44 | 0,53 | LOVD, ClinVar- likely benign |
| 19 | BRC102 | *MSH6* | 2 | het | Splice site variant | c.4001+12_4001+15delACTA |  | rs267608132 | 0 | 0,13 | 0 | LOVD, ClinVar- likely benign, VUS |

Abbreviations: VUS, variants of uncertain significance.
